# AI-guided spatiotemporal dispersion mapping for individualized ablation in an all-comer cohort with atrial fibrillation

**DOI:** 10.1007/s10840-025-02083-y

**Published:** 2025-06-06

**Authors:** Emanuel Heil, Nikolaos Dagres, Leif-Hendrick Boldt, Abdul Parwani, Florian Blaschke, Doreen Schoeppenthau, Philipp Attanasio, Robert Hättasch, Verena Tscholl, Gerhard Hindricks, Jin-Hong Gerds-Li, Felix Hohendanner

**Affiliations:** 1 Deutsches Herzzentrum Der Charité (DHZC), Charitéplatz 1, 10117 Berlin, Germany; 2 DZHK (German Centre for Cardiovascular Research), Partner Site Berlin, Berlin, Germany

**Keywords:** Atrial fibrillation, Spatiotemporal dispersion, Catheter ablation, Artificial intelligence, Substrate mapping

## Abstract

**Background:**

Artificial intelligence (AI)-guided spatiotemporal dispersion (stD) mapping has been shown to improve outcomes in patients with persistent atrial fibrillation (AF). However, the relationship between stD mapping and markers of atrial cardiomyopathy, dispersion patterns in paroxysmal versus persistent AF, stability of dispersion regions, and stD-guided ablation-related outcomes in all-comer cohorts remain elusive.

**Methods:**

In this retrospective single-center analysis, AF patients underwent high-density electroanatomical mapping alongside multiple instances of stD mapping using VOLTA AF Explorer software. Pulmonary vein isolation (PVI) and targeted ablation of left atrial dispersion regions were performed. Clinical, echocardiographic, biomarker, and low-voltage area (LVA) data were collected as markers of left atrial remodeling.

**Results:**

stD mapping identified dispersion in 92% of patients. Mean time since AF diagnosis was 7 ± 1 years. Overall, 58% of patients showed dispersion exclusively co-localizing with low-voltage areas, while 42% had dispersion extending into intermediate or normal voltage regions. Dispersion burden correlated strongly with LVA extent and other remodeling markers such as NT-proBNP and LAVI. Persistent AF patients exhibited a significantly higher number of dispersion sites compared to paroxysmal AF. Dispersion patterns remained largely consistent before and after cardioversion in persistent AF, with the posterior left atrial wall emerging as a common hotspot. At follow-up, AF recurred in 33% of paroxysmal and 60% of persistent AF patients who had dispersion ablation limited to the left atrium. Despite these recurrences, most patients reported an improvement in symptomatic burden.

**Conclusion:**

AI-guided stD mapping effectively identifies atrial remodeling beyond classical voltage-derived substrate, supporting its potential as a useful adjunctive tool in AF characterization.

**Graphical Abstract:**

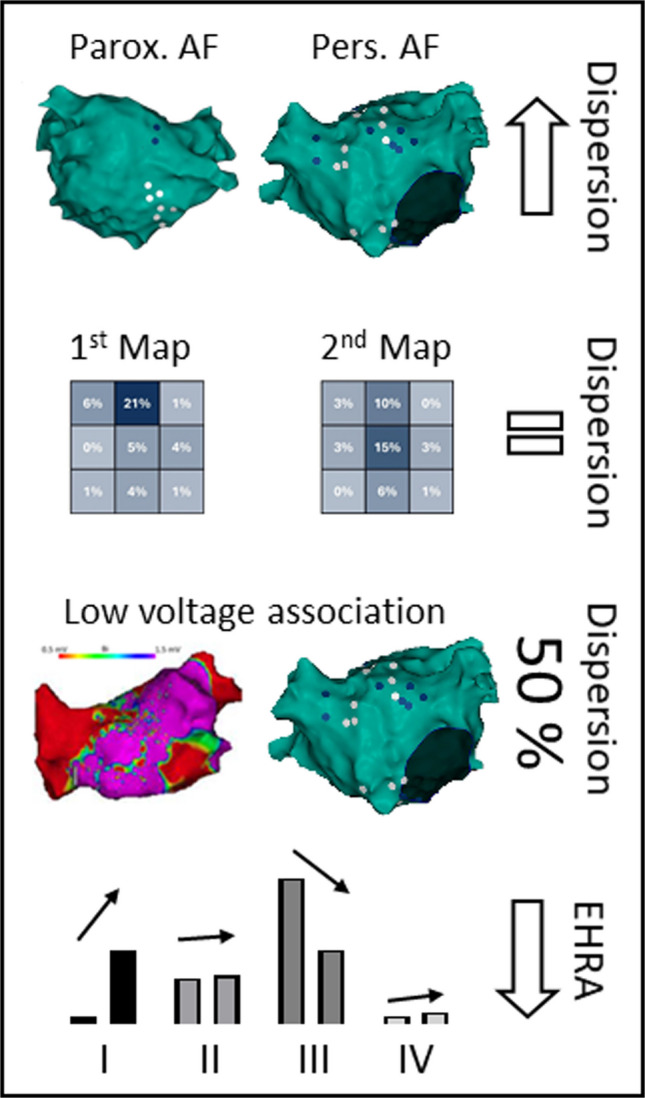

## Introduction

Atrial fibrillation (AF) is characterized by disorganized electrical activation and heterogeneous myocardial properties. A shared feature of fibrillatory arrhythmias is spatiotemporal dispersion, referring to spatial and temporal variability in conduction and refractoriness across the atria. Such dispersion of electrophysiological properties promotes wavefront fragmentation, reentrant circuit formation, and conduction block—key drivers for the initiation and maintenance of AF. While pulmonary vein ectopic beats act as triggers of AF [1], the persistence of AF, especially in advanced cases, is strongly influenced by the underlying atrial substrate. Regions of atrial cardiomyopathy or fibrosis can serve as anchors for reentrant circuits and sustain arrhythmia even after trigger elimination.

Pulmonary vein isolation (PVI) has become the cornerstone of AF ablation since Haïssaguerre et al.’s seminal 1998 report identifying pulmonary vein triggers [1]. In patients with paroxysmal AF, PVI alone is highly effective and is recommended as first-line therapy in current guidelines [2]. Long-term freedom from AF with an anatomical PVI-only approach is achieved in only approximately 50% of persistent AF patients. This limitation is attributed to the presence of diffuse electrical and structural remodeling in the atria (often termed atrial cardiomyopathy) that extends beyond the pulmonary vein focal triggers. Thus, additional ablation strategies targeting the atrial substrate have been extensively explored for improved outcomes in persistent AF.

Multiple substrate-based ablation approaches have been investigated, including linear lesion sets (e.g., left atrial roof or mitral isthmus lines), empirical isolation of the posterior left atrial wall, ablation of areas of delayed enhancement on MRI (fibrosis-guided ablation), and targeting of complex fractionated atrial electrograms (CFAEs) or focal/rotational drivers identified during AF [3, 4]. Unfortunately, large randomized trials have failed to demonstrate a consistent added benefit of these strategies over PVI alone [5, 6]. For example, the addition of left atrial lines or CFAE ablation did not significantly reduce AF recurrence in the STAR-AF II trial, and empirical posterior wall isolation or MRI-guided ablation has likewise shown no clear advantage [5–7]. One major challenge in these earlier substrate-directed approaches is the difficulty in objectively and reproducibly identifying the critical electrophysiologic substrate across different operators and centers. Voltage mapping of scar is a more objective marker of structural remodeling, and low-voltage area (LVA)-guided ablation has shown efficacy in some studies [8]. However, voltage alone provides a static view of the substrate and may miss dynamic conduction phenomena; moreover, not all trials of LVA-guided ablation have demonstrated benefit [9, 10].

Spatiotemporal dispersion mapping has emerged as a promising technique to fill this gap. Dispersion mapping involves analyzing high-density intracardiac electrograms during AF to identify localized regions where electrical activation is prolonged and fractionated over the AF cycle. In practical terms, stD is detected when clusters of adjacent bipoles on a multielectrode catheter record sequential activations spanning the full cycle length of AF [11], indicating an area of slow conduction and possible reentrant activity. Pioneering work by Seitz et al. demonstrated that visually identifying and ablating these dispersion regions could terminate AF and yield improved long-term outcomes [12]. In a study of 105 patients, a wholly patient-tailored ablation targeting dispersion (without initial PVI) acutely terminated AF in 95% of cases; at 18 months, 85% of patients remained arrhythmia-free with dispersion-guided ablation compared to 59% with a conventional stepwise ablation approach [13]. These findings suggested that the “electric footprint” of AF drivers could be effectively targeted via dispersion mapping, challenging the prevailing emphasis on anatomic lesion sets.

Building on these concepts, novel AI-driven mapping algorithms have been developed to automatically identify spatiotemporal dispersion in real time. The VOLTA Medical AF Explorer is one such software tool, trained on expert annotations of electrograms to detect dispersion objectively. This technology was recently tested in a large multicenter randomized trial, TAILORED-AF, which compared a tailored ablation strategy guided by AI-identified dispersion areas (in addition to PVI) against a standard PVI-alone strategy. The TAILORED-AF trial demonstrated a significantly higher single-procedure success rate at 12 months in the dispersion-guided ablation arm [11].

In light of these developments, we aimed to further characterize how spatiotemporal dispersion reflects the underlying atrial substrate in a real-world setting and how it might be used to guide ablation. Specifically, this pathophysiologic correlation study investigates the occurrence and distribution of stD in both paroxysmal and persistent AF patients, examines its relationship with established markers of atrial remodeling (such as low-voltage areas, atrial size, and biomarkers), and evaluates the stability of dispersion patterns during different recurring instances of AF episodes. By analyzing an all-comer cohort undergoing AF ablation with high-density mapping and AI-based dispersion detection, we sought to gain insights into the practical utility of stD mapping for substrate characterization. We also discuss how dispersion mapping fits into the broader context of substrate-based ablation strategies for AF and consider future directions for research and clinical application.

## Methods

This retrospective study included all patients (*n* = 26) who underwent catheter ablation for AF at our center in 2024 with adjunctive spatiotemporal dispersion mapping. Patients were included regardless of their rhythm history, with selection based on the availability of dispersion mapping. All patients provided written informed consent for the procedure, and the local ethics committee approved the study protocol (approval EA2_145_24).

The cohort consisted of paroxysmal and persistent AF cases, reflecting a mainly all-comer redo population. Each patient underwent high-density three-dimensional electroanatomical mapping of the left atrium using a multielectrode mapping catheter (PentaRay, Biosense Webster) and CARTO mapping system (Johnson & Johnson). In addition to standard voltage and activation mapping, AI-supported spatiotemporal dispersion (stD) mapping was performed using the VOLTA AF-Xplorer software (VOLTA Medical). This machine learning algorithm has been trained on expert-annotated intracardiac electrograms to identify regions of spatiotemporal dispersion. It analyzes signals recorded by the high-density mapping catheter (PentaRay in this study) and alerts the examiner when dispersion is detected. The corresponding segment is then annotated on the electroanatomical map reconstructed by the mapping system (CARTO in this study). This approach enables the AF-Xplorer system to remain compatible with various mapping platforms.

Since current evidence supports RA ablation primarily as an adjunct to LA procedures in selected patients with demonstrated right-sided drivers or complex failed prior LA ablation and empirically no patient in our cohort fulfilled these criteria, no RA ablation procedure was clinically indicated for the patients included in this retrospective investigation.

Baseline clinical data were collected for all patients, including age, sex, and relevant comorbidities. Echocardiographic parameters such as left ventricular ejection fraction (LVEF) and left atrial volume index (LAVI) were measured, and in a subset, global longitudinal strain was assessed. Markers of atrial cardiomyopathy were evaluated where available: N-terminal pro–B-type natriuretic peptide (NT-proBNP) levels, invasively measured left atrial pressure (LAP) at the start of the procedure, and left atrial low-voltage area (LVA) burden on voltage mapping. LVA burden was defined as the percentage of the left atrial surface area displaying low bipolar voltage (using a standard threshold for scar).

### Mapping protocol for paroxysmal vs persistent AF

**Fig. 1:**
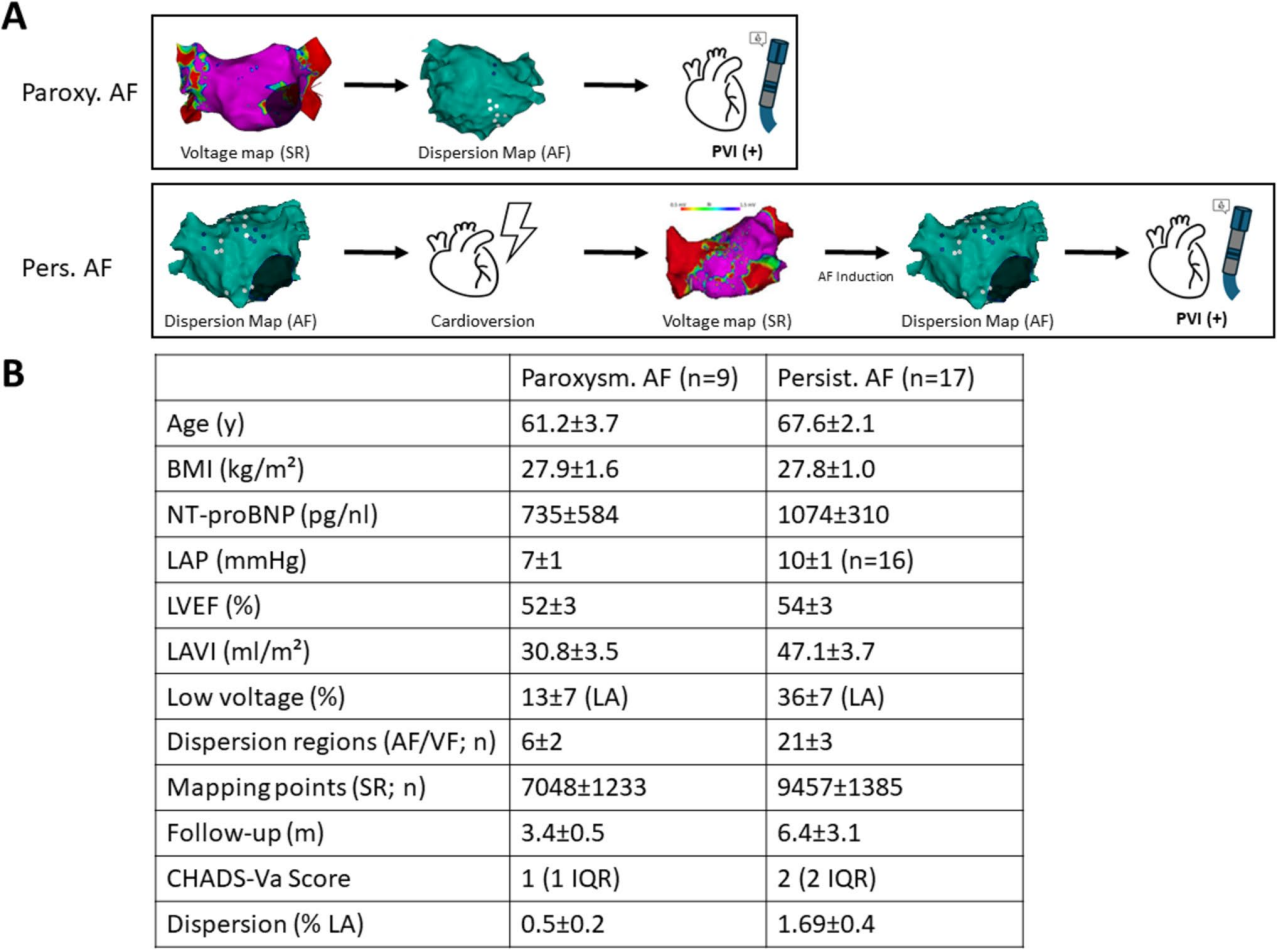
**A** Protocol used for voltage and dispersion mapping and ablation with RF-energy in patients with paroxysmal and persistent atrial fibrillation. **B** Retrospective clinical characteristics of the respective patient groups

Because the approach to mapping differed based on the AF type, two mapping protocols were employed (Fig. 1):

- Paroxysmal AF: In patients presenting in sinus rhythm at the start of the procedure, we first performed baseline voltage and activation mapping of the left atrium. AF was then induced using programmed atrial stimulation. During induced AF, a high-density map was acquired and analyzed with the dispersion mapping algorithm to identify regions of spatiotemporal dispersion. Based on the combined information from voltage mapping and dispersion mapping, ablation was carried out with pulmonary vein isolation as the foundation. If dispersion regions were identified outside the pulmonary vein antra, targeted ablation limited to these regions was performed after PVI. No empiric linear lesions (such as roof or mitral lines) were added in paroxysmal cases unless clinically indicated by inducible atrial tachycardia.
- Persistent AF: In patients with persistent AF, initial mapping was performed during ongoing AF to identify dispersion areas. A high-density AF map was created, and stD annotations were recorded. The patient was then cardioverted to sinus rhythm to allow assessment of the static substrate: a sinus rhythm voltage map of the left atrium was obtained to delineate scar areas. Thereafter, AF was re-induced by atrial pacing, and a repeat dispersion mapping was conducted during this re-induced arrhythmia. By comparing the dispersion findings from the initial (pre-cardioversion) AF map and the AF remap, we could evaluate the consistency of dispersion regions. Specifically, regions that showed *full dispersion* on both maps were considered stable dispersion sites. Ablation in persistent AF patients consisted of PVI (with reisolation of any reconnected veins) followed by targeted ablation at sites demonstrating persistent dispersion on both the initial and remap AF maps. As with paroxysmal AF, no additional non-PV linear lesions were empirically placed.

During stD mapping, we marked full dispersion sites blue and intermediate dispersion white in the electroanatomic map. For quantification, we estimated the *dispersion burden* as the fraction of the atrial surface area occupied by dispersion annotations. Based on the inter-electrode spacing of the PentaRay catheter, each dispersion annotation was assumed to represent approximately 10 mm^2^ of atrial tissue. The total number of dispersion annotations (blue tags) was summed and multiplied by 10 mm^2^ and then divided by the total mapped surface area to yield a percentage of atrial area exhibiting dispersion. This provides a quantitative measure to compare the dispersion extent between patients and between mapping phases.

All ablation lesions were delivered with an irrigated radiofrequency (RF) catheter (QDot Micro or Thermocool SmartTouch SF, Biosense Webster) following standard power and duration settings as per operator judgment. Pulmonary vein isolation was confirmed in all patients (entrance and exit block). Thereafter, RF applications were limited to full dispersion-positive regions (marked as blue spots): in paroxysmal AF, dispersion was mapped once during the induced arrhythmia and those regions were ablated; in persistent AF, only those regions that were consistently identified as full dispersion on both the initial and reinduced AF maps were targeted. The rationale was to focus on stable dispersion sites likely representing fixed substrate while avoiding chasing transient findings. Point-by-point ablation lesions were placed to cover the localized dispersion area, typically by creating a small cluster of lesions overlapping the dispersion annotations. Segments with overlapping LVA and STD were treated uniformly. Areas of intermediate dispersion (marked as white spots) were not ablated to avoid inducing atrial tachycardia circuits.

### Follow-up and outcome assessment

After the ablation, patients were followed in our clinic at regular intervals and additionally if symptoms occurred. Follow-up included clinical evaluation, 12-lead electrocardiograms, and 7-day Holter monitoring where feasible, in accordance with contemporary guidelines for AF ablation follow-up. AF recurrence was defined as any atrial fibrillation, flutter, or atrial tachycardia episode. Recurrences could be detected on Holter, ECG, or symptomatic event recordings. Arrhythmia recurrence and the need for repeat ablation were documented. Symptom burden was assessed using the European Heart Rhythm Association (EHRA) symptom classification, comparing pre-ablation and latest follow-up status. Antiarrhythmic medications were continued or initiated post-ablation at the physician’s discretion.

### Statistical analysis

Continuous variables were reported as mean ± standard error of the mean, and ordinal or non-normally distributed variables were given as median with interquartile range (IQR). Categorical data were expressed as percentages. Levene’s and *F* tests were used to evaluate equality of variances. Differences in interval-scaled subgroups were assessed with *t*-tests, Mann–Whitney *U* tests, Kruskal–Wallis, or Jonckheere–Terpstra tests, as appropriate, and Bonferroni correction was applied for multiple comparisons. Chi-square and Fisher’s exact tests were used for nominal and ordinal variables to detect deviations; Monte Carlo simulation was employed when needed to account for small sample sizes or distributional concerns. Correlation analyses were performed according to data distribution, following Cohen’s thresholds for effect size interpretation: small (*r* = 0.10), medium (*r* = 0.30), and large (*r* = 0.50). A *p*-value of ≤ 0.05 was considered statistically significant.

All statistical analyses were conducted with SPSS Statistics for Windows (version 29.0; IBM Corp., Armonk, NY), GraphPad Prism (version 10.4.1; GraphPad Software Inc., Boston, MA), and Microsoft 365 Excel Apps for Enterprise (version 2410; Microsoft, Redmond, WA).

## Results

### Patient characteristics and mapping findings

A total of 26 patients (mean age 66 ± 2 years, 23% female) underwent AF ablation with adjunct dispersion mapping. The cohort included nine patients with paroxysmal AF and 17 with persistent AF. The majority (≈77%) had undergone at least one prior AF ablation procedure before inclusion in this study, reflecting a population enriched in recurrence and remodeled substrate. The mean duration of AF history was 7 ± 1 years. Left ventricular systolic function was relatively preserved on average (LVEF 53 ± 2%), while markers of atrial remodeling were common: the mean left atrial volume index (LAVI) was 43 ± 3 mL/m^2^, the mean left atrial map volume 145 ± 7 mL, and the median CHA_2_DS_2_-VASc score was 2 (IQR 1–3). Of 22 patients with available labs, the median NT-proBNP was 754 pg/mL (IQR 224–1638), indicative of elevated atrial stress in many patients. Invasively measured left atrial pressure (LAP, available in 12 patients) averaged 9 ± 1 mmHg (Fig. 1).

High-density mapping was accomplished with an average of 4389 ± 669 mapping points acquired per patient. The mean overall procedural time was 120 ± 5 min, with a mean mapping time of 63 ± 22 min. Spatiotemporal dispersion was successfully detected in 24 out of 26 patients (92%) during the procedure. On the dispersion maps, an average of 17 ± 3 *full-dispersion* annotations were identified per patient, corresponding to an approximate stD burden of 1.4 ± 0.3% of the left atrial surface area. No relevant correlation existed between mapping density and dispersion annotation (*τ*_*b* = 0.232, *p* = 0.117). Left atrial volume quantified using 3D electroanatomic maps (*ρ* = 0.703, *p* < 0.001) and the area of the left atrial wall (*ρ* = 0.637, *p* < 0.001) strongly correlated with left atrial volume from preprocedural echocardiography.

Notably, two patients had *no* left atrial dispersion detected despite extensive mapping (mean 6958 ± 5388 points in those maps). These two patients with no stD had markedly lower indicators of atrial disease: their NT-proBNP levels were near-normal (98 ± 10 pg/mL, compared to the cohort mean > 1000 pg/mL; *p* = 0.043) and their LVA burden was minimal (1 ± 1% vs 30 ± 6% in those with any dispersion, *p* = 0.154). At follow-up, no recurrence was observed after pulmonary vein isolation.

Among the 24 patients with detectable dispersion, the spatial relationship between dispersion areas and low-voltage areas varied. In 14 patients (58% of the cohort, including 4 with paroxysmal and 10 with persistent AF, *concurrent* group), all dispersion annotations were located *within* regions of low voltage (< 0.5 mV). In the remaining 10 patients (42% of the cohort, including with 3 paroxysmal and with 7 persistent AF, *non-concurrent* group), dispersion sites were found not only in scarred low-voltage areas but also in regions of intermediate or normal voltage.

**Fig. 2:**
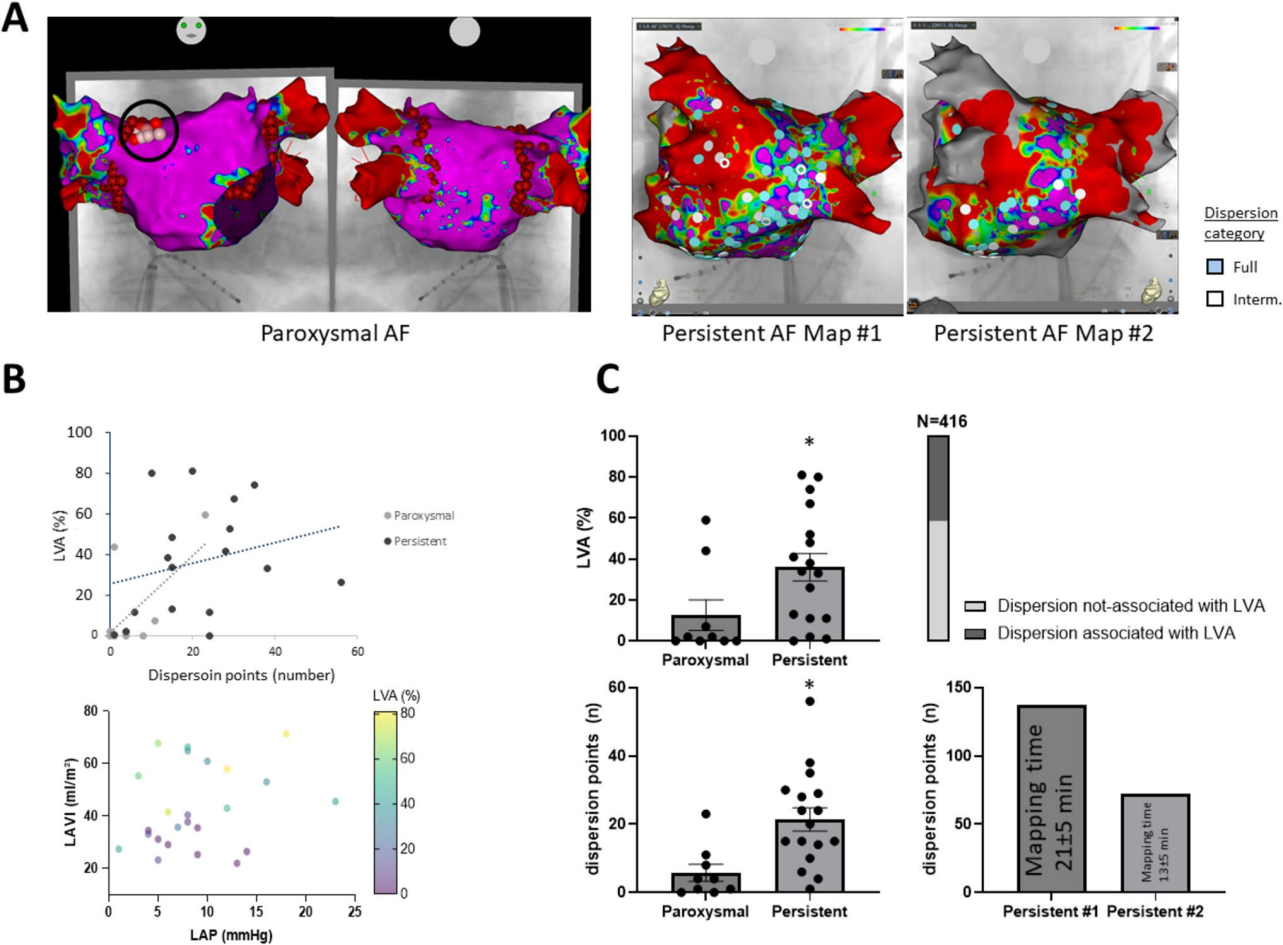
**A** 3D electroanatomic mapping (SR) example of paroxysmal (left) and persistent AF patients. Dispersion highlighted by black circle (full dispersion) for paroxysmal and white/blue points for persistent AF. **B** Quantitative analysis of dispersion points and left atrial low voltage areas in paroxysmal and persistent AF patients (top) as well as relation between LAVI and LAP in the cohort stratified by % of LVA (bottom). **C** Quantification of LVA and dispersion points in paroxysmal and persistent AF (left). Association of dispersion with LVA and mapping time for persistent AF dispersion map #1 and dispersion map #2 (right)

Figure 2 qualitatively illustrates examples of these patterns. When comparing these two subgroups, there were notable differences in atrial remodeling markers. Patients in the concurrent group (dispersion confined to LVA) had significantly larger left atria and more fibrosis than those in the non-concurrent group. Specifically, the concurrent group had a higher LVA burden (46 ± 7% vs 9 ± 4%, *p* < 0.001) and higher LAVI (49.4 ± 4.1 vs 33.8 ± 3.6 mL/m^2^, *p* = 0.011) than the non-concurrent group. Their NT-proBNP was also higher on average (1513 ± 541 pg/mL vs 437 ± 129 pg/mL; *p* = 0.05). Other clinical characteristics were similar between the two groups: there were no significant differences in age (66.8 ± 2.3 vs 64 ± 3.6 years), sex distribution, body mass index (27.5 ± 1.4 vs 28.5 ± 1.0 kg/m^2^), AF duration (median ~ 6 years in both), or number of prior ablations. These findings suggest that patients with more extensive atrial disease (large scar burden and dilation) tend to exhibit dispersion predominantly within scarred tissue. In contrast, patients with relatively healthier atria can still manifest dispersion phenomena in apparently normal-voltage regions.

**Fig. 3:**
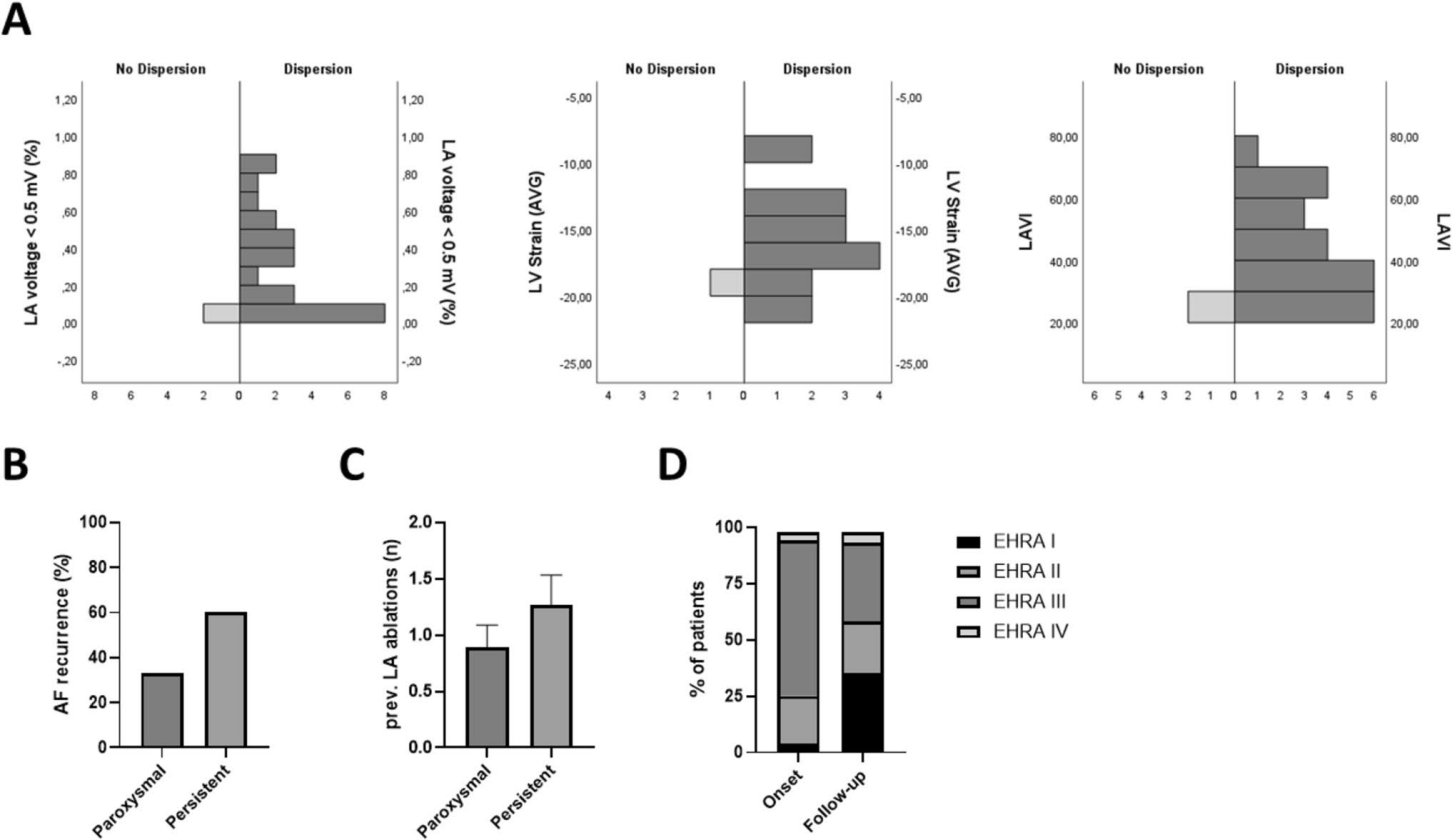
**A**. Quantitative distribution of LVA (i.e. LA voltage 0.5 mV), LV strain and LAVI in no dispersion vs dispersion positive patients. **B**. AF recurrence after PVI(+) in paroxysmal and persistent AF patients. **C**. Number of previous LA ablations in the cohort of paroxysmal and persistent AF patients. **D**. % of patients reporting the respective EHRA classification symptoms before ablation and at the end of retrospective follow-up

Across the entire cohort, dispersion burden showed strong associations with established markers of atrial cardiomyopathy (Fig. 3A). The percentage of the atrium exhibiting dispersion correlated positively with LVA percentage (*τ*_*b* = 0.419, *p* = 0.004). Patients with greater scar extent generally had more extensive dispersion. Additionally, higher dispersion burden was associated with higher NT-proBNP (*τ*_*b* = 0.519, *p* < 0.001) and larger LAVI (*τ*_*b* = 0.382, *p* = 0.007), indicating that dispersion mapping reflects underlying structural and functional remodeling of the atrium. We also observed that older age was moderately correlated with higher dispersion burden (*τ*_*b* = 0.355, *p* = 0.013), consistent with age-related progression of atrial fibrosis (indeed, age correlated with LVA, *τ*_*b* = 0.392; *p* = 0.006). In contrast, dispersion burden did not significantly correlate with acute hemodynamic measurements like LAP (*τ*_*b* = 0.136, *p* = 0.358) or with LVEF (*τ*_*b* = − 0.145, *p* = 0.323) or BMI (*τ*_*b* = − 0.200, *p* = 0.157). These results reinforce that stD is a robust marker of the underlying atrial substrate—closely tracking fibrosis, atrial size, and atrial stretch biomarkers—rather than an epiphenomenon of acute loading conditions or ventricular function.

### Paroxysmal vs persistent AF: dispersion burden and distribution

**Fig. 4:**
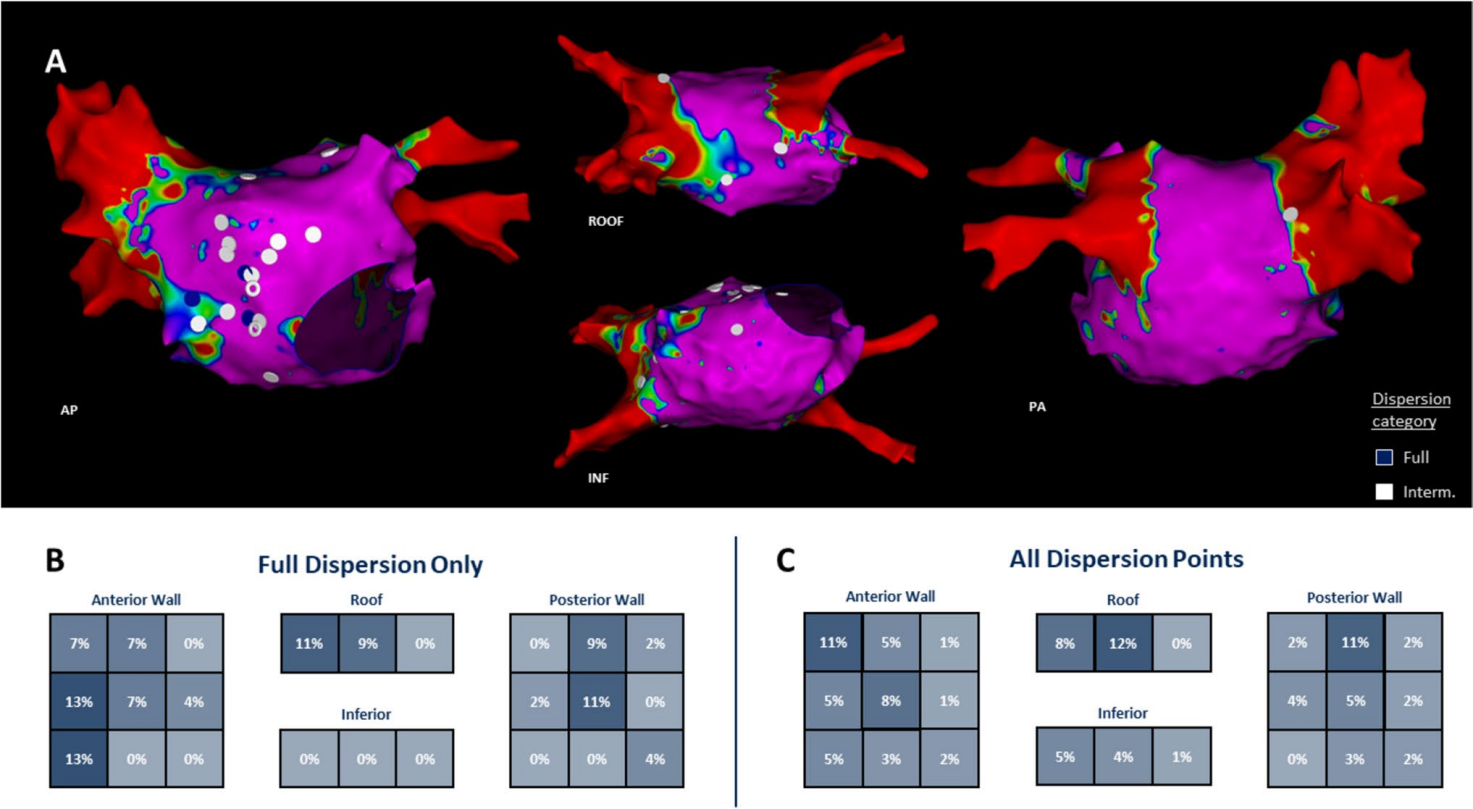
**A**. Example 3D electroanatomic map of a patient with paroxysmal AF, blue and white dots highlight regions of full and intermediate dispersion, respectively. Distribution of full dispersion (**B**) and all dispersion points (**C**) in patients with paroxysmal AF, blue and white dots highlight regions of full and intermediate dispersion, respectively

Next, we analyzed dispersion characteristics separately in the paroxysmal and persistent AF subsets (Figs. 2 and 3 for paroxysmal AF and Figs. 2 and 4 for persistent AF). Among the nine patients with paroxysmal AF, the mean age was 61 ± 4 years, and 78% were male. Echocardiography showed relatively smaller atria (mean LAVI ~ 31 mL/m^2^) and normal left ventricular function (LVEF 52 ± 3%). Most paroxysmal AF patients had a history of prior ablation (78% with at least one prior PVI). On high-density mapping (average 7048 ± 1233 points per map), we found a modest dispersion burden: on average 6 ± 2 full-dispersion sites per patient, corresponding to 0.5 ± 0.2% of the atrial area. In these paroxysmal cases, dispersion sites tended to cluster in particular anatomical regions (Fig. 3). The septal wall and the superior aspect of the posterior left atrial wall (the upper-middle posterior wall) were identified as common *hotspots* for dispersion (i.e., sites where multiple patients had dispersion annotations). This suggests that even in paroxysmal AF, there are preferential locations for conduction heterogeneity, possibly related to fiber orientation or transitional tissue in those areas. However, overall dispersion in paroxysmal AF was limited in extent, and in some patients, only a few small areas of dispersion were present (with some paroxysmal patients having none or only intermediate dispersion, as noted above).

Substrate remodeling was more pronounced in the 17 patients with persistent AF (Fig. 3). Persistent AF patients were older (mean 68 ± 2 years, vs 61 ± 4 in paroxysmal, *p* = 0.107) and had larger atria (LAVI 47.1 ± 3.7 vs 30.8 ± 3.5 mL/m^2^, *p* = 0.006). They also had significantly higher NT-proBNP levels (1074 ± 310 vs 735 ± 584 pg/mL, *p* = 0.021), reflecting greater atrial strain. Other factors such as BMI (~ 28 kg/m^2^ in both), CHADS-VASc score (median 2 vs 1, *p* = 0.178), and LVEF (~ 54% vs ~ 52%, *p* = 0.426) were similar between persistent and paroxysmal groups. All but one persistent AF patient had prior ablation(s) (76% with at least one PVI; one patient had an extensive substrate ablation previously). High-density AF mapping was performed in all persistent cases (mean 9457 ± 1385 points per map, not significantly different from paroxysmal mapping density, *p* = 0.458). As expected, the extent of low-voltage substrate was larger in persistent AF (mean LVA 36 ± 7% of LA, vs 13 ± 7% in paroxysmal, *p* = 0.021; Fig. 2C). Correspondingly, the number of dispersion sites was markedly higher: persistent AF patients had on average 21 ± 3 dispersion annotations per map, nearly fourfold greater than paroxysmal patients (*p* = 0.001). This translated to a higher total dispersion burden of 1.69 ± 0.4% of the atrium affected by dispersion in persistent AF, compared to 0.5 ± 0.2% in paroxysmal AF (*p* = 0.006). Thus, patients with persistent AF had more scar and disproportionately more dispersion, suggesting that ongoing AF and atrial remodeling create a more heterogeneous electrical substrate.

Qualitatively, dispersion in persistent AF was more widespread across the atrium, but some preferential clustering was still demonstrated. Like the paroxysmal group, the *posterior left atrial wall* (particularly the upper-middle segment near the left pulmonary veins) was frequently involved with dispersion in persistent AF patients (Fig. 4). Many persistent AF maps showed a band of dispersion tags along the posterior wall and extending to adjacent regions such as the posterior septum or the junction of the posterior wall with the roof. Additionally, dispersion sites in persistent AF often coincided with border zones of scar defined as areas where voltage was intermediate between dense scar and healthy tissue.

### Stability of dispersion mapping after cardioversion (remapping in persistent AF)

In six of the persistent AF patients, we performed repeat mapping. This subset (mean age 66 ± 4 years, LAVI 41 ± 3 mL/m^2^, LVA 28 ± 8%) had substantial atrial disease but represented the broader persistent group. The initial dispersion map in these patients (acquired during pre-cardioversion AF, with mapping time ~ 21 ± 5 min and 7258 ± 1703 points collected) showed a broad distribution of dispersion sites across the left atrium. After cardioversion and reinducing AF (remap mapping time ~ 13 ± 5 min, 4100 ± 1702 points), the overall number of dispersion annotations per patient decreased (from an average of 24 on the initial map to 14 on the remap; Figs. 2A and 4). Despite this reduction in count, the *spatial pattern* of dispersion remained similar in many respects. This indicates that at least a subset of dispersion regions are stable features of the substrate, persisting even after interruption of AF and its reinitiation. The more uniform dispersion distribution on the remap (as opposed to the initial map which sometimes had more clustered dispersion) could be due to changes in AF cycle length or organization after cardioversion, but this requires further investigation. Interestingly, three patients in the remapping subset demonstrated *new* dispersion areas on the remap that were not evident on the initial map. These newly emerged sites did not show any clear relationship with procedural or physiological changes.

### Clinical outcomes

Over a mean follow-up of 5.3 ± 2.2 months (range ~ 3 to 9 months) after the ablation procedure, arrhythmia recurrence and symptom status were assessed in the 24 patients who had dispersion-guided ablation (2 patients with no dispersion had only PVI and remained in sinus rhythm). Atrial fibrillation recurrence was documented in 12 of 24 patients (50%). When stratified by AF type, 3 out of 9 (33%) paroxysmal AF patients experienced, whereas 9 out of 15 (60%) persistent AF patients had at least one arrhythmia recurrence during follow-up.

**Fig. 5:**
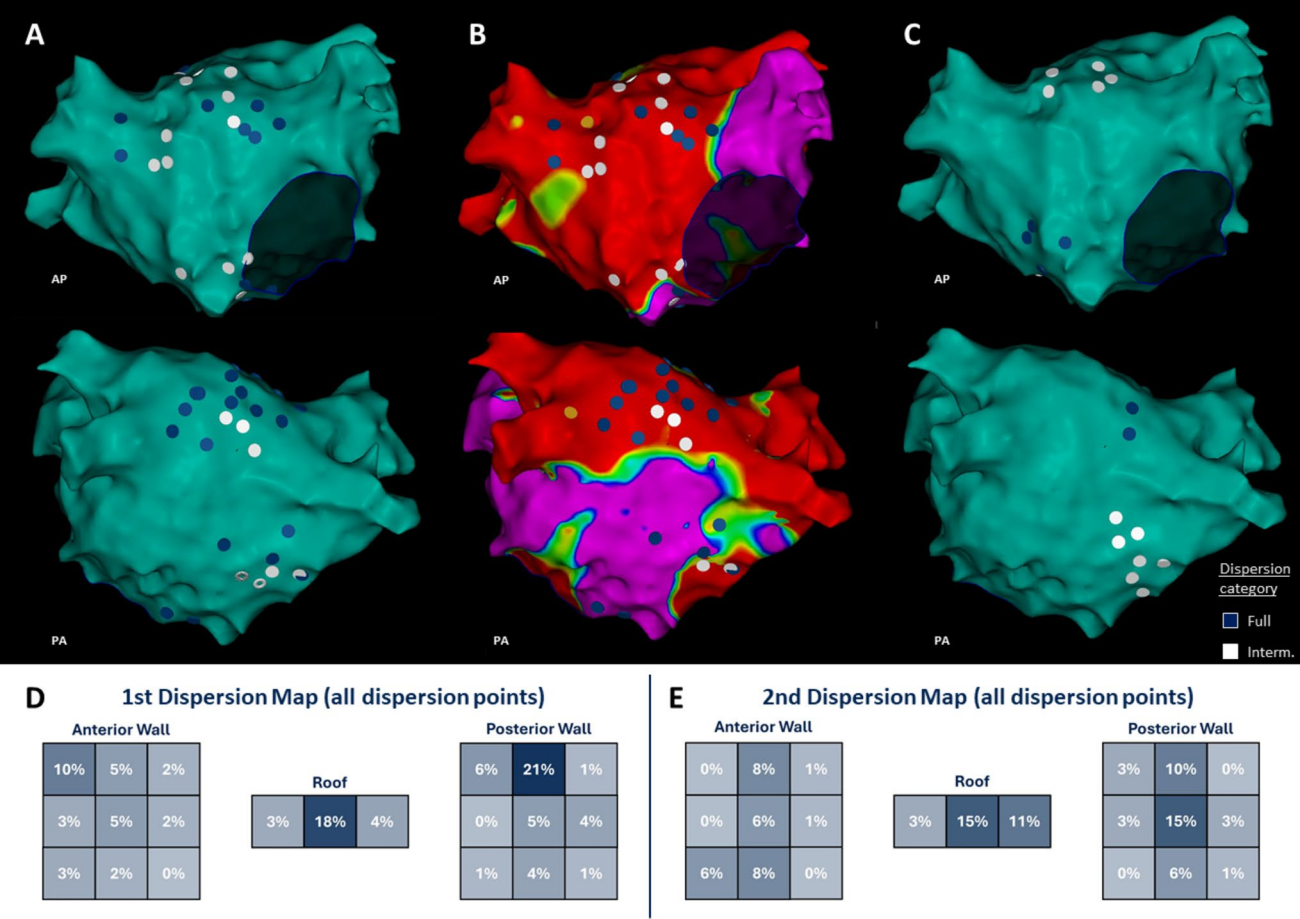
Example distribution of all dispersion points in a patient with persistent AF during the 1st (**A**) and 2nd (**C**) dispersion map. **B**. Voltage map in the example patient. **D** and **E**. Quantitative distribution of dispersion points at the anterior wall, the roof and the posterior wall in all patients with persistent atrial fibrillation during 1st and 2nd dispersion maps, blue and white dots highlight regions of full and intermediate dispersion, respectively

Importantly, the *symptom burden* improved in the majority of patients, including many of those with recurrence. Based on EHRA class assessment, 20 of 24 patients (83%) reported a reduction in AF-related symptoms compared to their pre-ablation status (pre-ablation EHRA 3 (1 IQR) vs post-ablation median EHRA 1.5 (3 IQR)). Figure 5B and D illustrate the shift in symptom class). Many patients moved from EHRA Class III (significant limitations) to Class I–II (mild or no symptoms) after ablation. No strokes, tamponades, atrioesophageal fistula, or other major complications occurred in this cohort during follow-up.

## Discussion

This study demonstrates that AI-guided spatiotemporal dispersion mapping effectively identifies electrophysiological heterogeneity associated with AF substrate beyond conventional mapping techniques. Our key findings are that dispersion burden correlates significantly with markers of structural atrial remodeling, is notably higher in persistent compared to paroxysmal AF, and frequently localizes to border zones of low-voltage scar tissue.

Despite substantial advances, outcomes for persistent AF remain suboptimal with pulmonary vein isolation alone. Consequently, substrate-based strategies have been widely explored but have yielded inconsistent results [4]. Techniques such as complex fractionated electrogram ablation, rotor ablation, and linear lesion strategies have struggled to demonstrate superiority over PVI in randomized trials due to subjective interpretation, variability, and procedural complexity [3–6].

Spatiotemporal dispersion mapping, particularly when AI-driven, addresses several limitations inherent in earlier substrate-based approaches. By objectively identifying prolonged activation regions indicative of conduction slowing or reentry, dispersion mapping offers reproducibility across operators and centers. This is underscored by the recently published TAILORED-AF trial, which demonstrated superior single-procedure freedom from AF at 1 year (88% vs 70%, *P* < 0.0001) using extensive left and right atrial dispersion-guided ablation alongside PVI. However, TAILORED-AF’s comprehensive ablation strategy significantly increased procedural duration, suggesting a need to balance efficacy with procedural feasibility [11]. In line with this notion, initial mapping times in the persistent cohort of our study were at least 10 min shorter than those in TAILORED-AF, and ablation was limited to stable full-dispersion regions.

In our patients, dispersion burden correlated strongly with traditional markers of atrial cardiomyopathy, including atrial size, fibrosis extent (low-voltage area, LVA), and biomarkers (NT-proBNP). The absence of left atrial dispersion in the two patients without evidence of atrial cardiomyopathy strongly suggests that dispersion may serve as a promising additional marker of atrial remodeling beyond mere voltage.

Importantly, dispersion was frequently found in intermediate-voltage areas, highlighting regions of active conduction heterogeneity potentially missed by voltage mapping alone. Persistent AF patients exhibited substantially higher dispersion burden and more extensive remodeling, reflecting the greater complexity and advanced substrate in these patients. This notion is supported by previous reports showing dispersion positive regions in close vicinity to the underlying substrate of a patient with atrial surgery related scar [14]. In our cohort, which included 77% of patients with prior ablation, dispersion hotspots were consistently observed on the posterior left atrial wall and at scar border zones. These hotspots roughly correspond to the expected locations of atrial ganglionated plexi, but more importantly, they overlap with common target areas of pulmonary vein isolation. This aligns with previous evidence suggesting that incomplete ablation scars can promote a proarrhythmic substrate [15]. This finding allows for the hypothesis that dispersion mapping may help identify iatrogenic atrial fibrillation triggers following prior pulmonary vein isolation. Future studies should build on this important insight, as high-density mapping, including dispersion analysis, is particularly relevant for redo cases, especially given the increasing proportion of primary pulmonary vein isolations performed with single-shot devices.

Our remapping approach in persistent AF patients also demonstrated that certain dispersion sites remain stable and reproducible, whereas others may appear transiently after rhythm restoration. Therefore, targeting stable dispersion areas could offer a more efficient approach to substrate ablation, potentially enhancing outcomes while minimizing unnecessary ablation. The study protocol did not allow for assessing the impact of multiple cardioversions.

Our overall single-procedure success rate (~ 50%) was consistent with expected outcomes for a challenging, predominantly redo ablation population [16–18]. While lower than TAILORED-AF’s outcomes, this likely reflects differences in patient complexity (redo), lesion extent (more substate), and ablation strategy (no lines, left atrial dispersion only). Nonetheless, significant symptom improvement (83% reporting improved EHRA class) highlights clinical benefit even in patients with “AF recurrence.” The discrepancy between recurrence rates and observed clinical benefit may be explained by an overall reduction of AF burden despite the formal occurrence of recurrent tachyarrhythmias, improved medical therapy, placebo effects, or reporting bias.

This study’s primary aim related to a better understanding of dispersion in the context of AF, yet its major limitations include its retrospective, single-center nature, modest cohort size with small subgroups, and absence of a contemporaneous control group. Short-term follow-up limits assessment of sustained clinical benefit, and importantly systematic right atrial dispersion mapping was not performed. Future prospective trials should evaluate the efficacy of left and right dispersion mapping-based ablation in patients undergoing redo procedures for atrial fibrillation, comparing this approach with established ablation strategies.

In conclusion, AI-guided dispersion mapping appears promising for individualized ablation therapy in AF. Objectively identifying critical substrate regions complements traditional anatomical and voltage-based approaches. Balancing procedural complexity with targeted lesion delivery may ultimately maximize efficacy, safety, and clinical outcomes.

## Declaration of generative AI and AI-assisted technologies in the writing process

During the preparation of this work, the authors used ChatGPT 4.5 to improve language. After using this tool/service, the authors reviewed and edited the content as needed and take full responsibility for the publication’s content.

## Data Availability

The data underlying this article will be shared on reasonable request to the corresponding author.

## Abbreviations

AF: Atrial fibrillation
LAVI: Left atrial volume index
LVA: Low-voltage area
PVI: Pulmonary vein isolation
stD: Spatiotemporal dispersion
